# The Impact of Gut Microbiota on Gender-Specific Differences in Immunity

**DOI:** 10.3389/fimmu.2017.00754

**Published:** 2017-06-30

**Authors:** Floris Fransen, Adriaan A. van Beek, Theo Borghuis, Ben Meijer, Floor Hugenholtz, Christa van der Gaast-de Jongh, Huub F. Savelkoul, Marien I. de Jonge, Marijke M. Faas, Mark V. Boekschoten, Hauke Smidt, Sahar El Aidy, Paul de Vos

**Affiliations:** ^1^Top Institute Food and Nutrition, Wageningen, Netherlands; ^2^Department of Pathology and Medical Biology, University Medical Center Groningen, University of Groningen, Groningen, Netherlands; ^3^Cell Biology and Immunology Group, Wageningen University, Wageningen, Netherlands; ^4^Laboratory of Microbiology, Wageningen University, Wageningen, Netherlands; ^5^Laboratory of Pediatric Infectious Diseases, Radboud University Medical Center, Nijmegen, Netherlands; ^6^Department of Obstetrics and Gynaecology, University Medical Center Groningen, University of Groningen, Groningen, Netherlands; ^7^Nutrition, Metabolism and Genomics Group, Division of Human Nutrition, Wageningen University, Wageningen, Netherlands; ^8^Microbial Physiology, Groningen Biomolecular Sciences and Biotechnology Institute, University of Groningen, Groningen, Netherlands

**Keywords:** gender, gut microbiota, germ-free mice, immunity, inflammation

## Abstract

Males and females are known to have gender-specific differences in their immune system and gut microbiota composition. Whether these differences in gut microbiota composition are a cause or consequence of differences in the immune system is not known. To investigate this issue, gut microbiota from conventional males or females was transferred to germ-free (GF) animals of the same or opposing gender. We demonstrate that microbiota-independent gender differences in immunity are already present in GF mice. In particular, type I interferon signaling was enhanced in the intestine of GF females. Presumably, due to these immune differences bacterial groups, such as *Alistipes, Rikenella*, and Porphyromonadaceae, known to expand in the absence of innate immune defense mechanism were overrepresented in the male microbiota. The presence of these bacterial groups was associated with induction of weight loss, inflammation, and DNA damage upon transfer of the male microbiota to female GF recipients. In summary, our data suggest that microbiota-independent gender differences in the immune system select a gender-specific gut microbiota composition, which in turn further contributes to gender differences in the immune system.

## Introduction

It is widely accepted that there are differences in the immune system between males and females. Males are generally more susceptible to infections (1), whereas prevalence of various autoimmune disorders is much higher in females (2, 3). Different concentrations of sex steroids, such as testosterone, estrogens, and progesterone, could contribute to these immune differences, since sex steroids can influence the function of immune cells by binding to specific receptors expressed on these cells (1). Genetic differences between males and females are also considered to contribute, since the X chromosome is known to contain the largest number of genes involved in immunity of the whole genome (4, 5).

A third factor that could contribute to gender differences in the immune system is the gut microbiota. Our intestine harbors a highly complex community of bacteria, which is separated from a large pool of immune cells by only a single layer of epithelial cells (6). Consequently, gut microbiota composition shapes the immune system and *vice versa* (7). Several studies have shown that gut microbiota composition differs between males and females (8–11), which could potentially contribute to the observed gender-specific differences in immunity. The importance of these differences for gender-specific disease development has been shown in type 1 diabetes in which a higher prevalence of the disease in females was shown to be critically dependent on the gut microbiota (10, 11), demonstrating a link between gut microbes, gender, and immunity. However, whether gender-dependent immunity differences are a cause or consequence of an altered gut microbiota composition is not clear.

To determine the contribution of the gut microbiota to gender-specific differences in the immune system, in this study, we performed gut microbiota transfer experiments from conventional male or female mice to germ-free (GF) recipient mice of the same or opposing gender. Effects on the immune system were assessed by measuring genome-wide gene expression levels in the ileum and by analyzing different immune cell populations in the thymus, spleen, mesenteric lymph nodes (MLNs), and Peyer’s patches (PPs).

## Materials and Methods

### Mice

Female and male C57BL/6JRccHsd conventional 7- to 10-week-old mice were purchased from a commercial supplier (Envigo, Horst, the Netherlands). Female and male GF mice, 12- to 14-weeks old, were obtained from a breeding colony at the animal facility of Radboud University Nijmegen Medical Centre (Nijmegen, the Netherlands). All animals were put on an autoclaved rat/mouse maintenance V153X R/M-H diet (Ssniff, Soest, Germany) directly after weaning in the case of GF mice, or directly after arrival in the case of conventional mice. The mice were kept on this diet throughout the experiment. Conventional mice were housed in IVC cages and GF mice were housed in GF isolators. All experiments were approved by the local ethical committee of the University of Groningen.

### Experimental Design

After an acclimatization period of at least 4 weeks, feces were freshly collected from the conventional males (*n* = 8) or conventional females (*n* = 10). Feces from the same group was pooled and mixed in PBS. Next, 200 µl of 100 mg/ml of this mixture was given by oral gavage to age-matched GF mice of the same or opposing gender (*n* = 10 per experimental group). After transfer, recipient mice were individually housed in IVC cages for another 4 weeks. As a control, male and female GF mice (*n* = 5) were kept germ free throughout the experiment. In summary, there were the following experimental groups:

1. Conventional males (*n* = 8)
2. Conventional females (*n* = 10)
3. GF males that received microbiota from conventional males (*n* = 10)
4. GF females that received microbiota from conventional females (*n* = 10)
5. GF males that received microbiota from conventional females (*n* = 10)
6. GF females that received microbiota from conventional females (*n* = 10)
7. GF males as a control (*n* = 5)
8. GF females as a control (*n* = 5)

### Organ and Tissue Collection

Mice were sacrificed at the following ages: conventional mice 16–19 weeks, GF recipient mice 16–18 weeks, and GF mice 13–15 weeks. The GF control mice were slightly younger than the other groups of mice since it was logistically not feasible to obtain enough GF mice of the same age of both sexes from our breeding colony at the same time. Mice were anesthetized with isoflurane, bled, and sacrificed by cervical dislocation. Serum was collected and stored at −80°C. Colon content and a piece of terminal ileum were snap frozen in liquid nitrogen and stored at −80°C. In addition, spleen, thymus, PPs, and MLNs were collected for FACS analysis.

### Flow Cytometry

Single cell suspensions were obtained from spleen, thymus, PPs, and MLNs. Cells were stained with Fixable Viability Dye eFluor 506 (eBioscience, Vienna, Austria) for exclusion of dead cells. A-specific binding to Fc receptors was blocked by incubating the cells with anti-CD16/32 for 15 min on ice. For extracellular staining, cells were incubated with the desired mixture of antibodies for 30 min on ice. After washing, cells were fixed with FACS lysing solution (BD Biosciences, Breda, the Netherlands). For intracellular staining, fixed cells were permeabilized with PERM (eBioscience, Vienna, Austria) and subsequently stained with the desired antibodies for 30 min on ice. For identification of the different T helper (Th) cell subsets, cells were stained with antibodies against CD3e (clone 17 A2), CD4 (clone GK1.5), T-bet (clone 4B10), RORyt (clone B2D), Gata-3 (clone TWAJ), CD25 (clone PC61), and Foxp3 (clone FJK-16S). Appropriate isotype controls were used to determine specificity of the staining. T cell precursors in thymus were identified by excluding other lineages: CD11b (clone M1/70), CD11c (clone HL3), CD19 (clone 1D3), CD45R/B220 (clone RA3-6B2), NK1.1 (clone PK136), and TER119 (clone TER-119). Next, cells were gated on populations that were double negative (DN), single positive, or double positive for CD4 (clone GK1.5) and CD8a (clone 53-6.7). To identify earliest T cell precursors (triple negative), cells positive for CD3e (clone 145-2C11) were excluded. Samples were acquired with the FACSVerse or FACSCanto II (BD Biosciences, Breda, the Netherlands) and analyzed with FlowJo software (FlowJo, LLC, OR, USA).

### Transcriptome Microarray

A piece of terminal ileum from each mouse was snap frozen in liquid nitrogen and stored afterward at −80°C. RNA was isolated with the RNeasy kit (Qiagen, Valencia, CA, USA). Quantity of RNA was measured with the ND-1000 (NanoDrop Technologies, Thermo Fisher Scientific, Breda, the Netherlands) and quality of RNA was assessed with the Bioanalyzer 2100 (Agilent, Santa Clara, CA, USA). Total RNA (100 ng) was labeled utilizing the Ambion WT Expression kit (Life Technologies Ltd., Bleiswijk, the Netherlands) and the Affymetrix GeneChip WT Terminal Labeling kit (Affymetrix, Santa Clara, CA, USA). After labeling, samples were hybridized to Affymetrix GeneChip Mouse Gene 1.1 ST arrays. An Affymetrix GeneTitan Instrument was used for hybridization, washing, and scanning of the array plates. Bioconductor packages integrated in an online pipeline were used for quality control of the data (12, 13). Probe sets were redefined using current genome information (14). Probes were reorganized based on the Entrez Gene database (remapped CDF v14.1.1). Robust Multi-array Analysis preprocessing algorithm available in the Bioconductor library affyPLM (15) was used to obtain normalized expression estimates from the raw intensity values.

### Serum Antibody Isotypes

Mouse serum samples were assayed for mouse IgG1, IgG2a, IgG2b, IgG3, IgA, IgE, and IgM using ProcartaPlex Mouse Antibody Isotyping Panel on the Luminex platform (Affymetrix, Santa Clara, CA, USA), according to manufacturer’s instructions. In brief, samples were thawed on ice. Beads were mixed and washed and subsequently incubated overnight at 4°C with standards or with 1:500 or 1:50,000 diluted samples. After washing, the beads were incubated with detection antibody mix for 30 min at room temperature. The beads were washed and incubated for 30 min at room temperature with streptavidin-PE. After washing the beads were measured with a Luminex instrument (Bio-Plex 200, Bio-Rad), which was calibrated using Bio-Rad calibration beads. Standard curves were calculated using 5-parameter logistic regression in Bio-plex 5.0 software.

### Microbiota Analysis

Fresh feces samples obtained just after defecation were collected from all mice at different time points during the experiment. In addition, colonic content samples from these mice were collected at the end of the experiment. All samples were snap frozen in liquid nitrogen and stored at −80°C. These samples were used for 16S rRNA gene analysis for microbiota profiling with barcoded amplicons from the V1–V2 region of 16S rRNA genes generated using a 2-step PCR strategy that reduces the impact of barcoded primers on the outcome of microbial profiling (16). DNA extraction was performed using a combination of the bead-beating-plus column method and the Maxwell 16 Tissue LEV Total RNA purification kit (Promega, Leiden, the Netherlands). Beating of the fecal pellets took place as described before (17), but with STAR (Stool transport and recovery) buffer (Roche, Basel Switzerland). 250 µl supernatant after centrifugation was taken for the Maxwell 16 Tissue LEV Total RNA Purification Kit, and the DNA was eluted in 50 µl DNAse-free water. Twenty nanograms of DNA were used for the amplification of the 16S rRNA gene with primers 27F-DegS and 338R I + 338R II for 25 cycles as described before (18), only primers had a Universal Tag (UniTag) linkers attached; UniTag I (forward) and II (reverses) (I—GAGCCGTAGCCAGTCTGC; II—GCCGTGACCGTGACATCG). The first PCR was performed in a total volume of 50 µl containing 1× HF buffer (Finnzymes, Vantaa, Finland), 1 µl dNTP Mix (10 mM; Promega, Leiden, the Netherlands), 1 U of Phusion^®^ Hot Start II High-Fidelity DNA polymerase (Finnzymes Vantaa, Finland), 500 nM of the 27F-DegS primer (18, 19) that was appended with UniTag 1 at the 5′ end, 500 nM of an equimolar mix of two reverse primers, 338R I and II (19) based on three previously published probes EUB 338 I, II, and III (18), that were 5′-extended with UniTag 2, and 0.2–0.4 ng/µl of template DNA. The sequence of the UniTags were selected to have a GC content of ~66% and a minimal tendency to form secondary structures, including hairpin loops, heterodimers, and homodimers as assessed by the IDTDNA Oligoanalyzer 3.1 (Integrated DNA Technologies). Moreover, sequences were selected that had no matches in 16S rRNA gene databases (based on results of the “TestProbe” tool offered by the SILVA rRNA database project (20) using the SSU r117 database), and no prefect matches in genome databases with the Primer-BLAST tool (http://www.ncbi.nlm.nih.gov/tools/primer-blast/). The size of the PCR products (~375 bp) was confirmed by gel electrophoresis using 5 µl of the amplification reaction mixture on a 1% (w/v) agarose gel containing 1× SYBR^®^ Safe (Invitrogen, Thermo Fisher Scientific, Waltham, MA, USA). Five microliters of these PCR products were taken to add adaptors and a 8-nt sample-specific barcode in an additional 5 cycle PCR amplification. This second PCR was performed in a total volume of 100 µl containing 1× HF buffer, dNTP Mix, 2 U of Phusion^®^ Hot Start II High-Fidelity DNA polymerase, 500 nM of a forward and reverse primer equivalent to the Unitag1 and UniTag2 sequences, respectively, that were each appended with an 8 nt sample-specific barcode (G. Hermes and J. Ramiro-Garcia, et al., in preparation) at the 5′ end. PCR products were purified with the magnetic beads (MagBio, London, UK) according to the HighPrepTM protocol of the manufactures instructions using 20 µl nuclease-free water (Promega Leiden, the Netherlands) and quantified using the Qubit (Life Technologies, Bleiswijk, the Netherlands). Purified PCR products were mixed in approximately equimolar amounts and concentrated by the magnetic beads as the purification before. Purified amplicon pools were 250 bp paired-end sequenced using Illumina Miseq (GATC-Biotech, Konstanz, Germany).

The Illumina Miseq data analysis was carried out with a workflow employing the Quantitative Insights Into Microbial Ecology (QIIME) pipeline (21) and a set of in-house scripts as described before for Illumina Hiseq 16S rRNA gene sequences (G. Hermes and J. Ramiro-Garcia, et al., in preparation). The set of in-house scripts processed the reads as follows: reads were filtered for not matching barcodes; OTU picking and chimera removal was done *via* matching the sequences to the Silva 111 database, with only one mismatch allowed, and a biom and with ClustalW a multiple alignment and phylogenetic tree file was generated. Further outputs were generated *via* QIIME, such as filtered reads per sample, PD whole tree diversity measurements and the level 1 to 6 taxonomic distributions with relative abundances.

### Statistics

Flow cytometry data and serum antibody isotype data are expressed as means. To verify whether data were normally distributed the Kolmogorov–Smirnov test was performed. In cases where data were not normally distributed, data were log transformed before analysis. For comparing two groups, the unpaired two-tailed Student’s *t*-test was used. For comparing more than two groups with each other, one way ANOVA was performed followed by the Bonferroni test to compare specific groups. *p*-Values below 0.05 were considered significant, and *p*-values below 0.1 were considered a trend. All tests were performed with GraphPad software (Prism, La Jolla, CA, USA).

Differentially expressed probe sets were identified using linear models, applying moderated T-statistics that implemented empirical Bayes regularization of SEs (22). A Bayesian hierarchical model was used to define an intensity-based moderated T-statistic, which takes into account the degree of independence of variances relative to the degree of identity and the relationship between variance and signal intensity (23).

Statistical tests for gut microbiota composition were performed using R and Calypso (24). Where the count data were not normally distributed and variances between groups were not equal, the Mann–Whitney *U* test was used.

## Results

### Male Microbiota Induces Lower Weight in Female Mice

**Figure 1:**
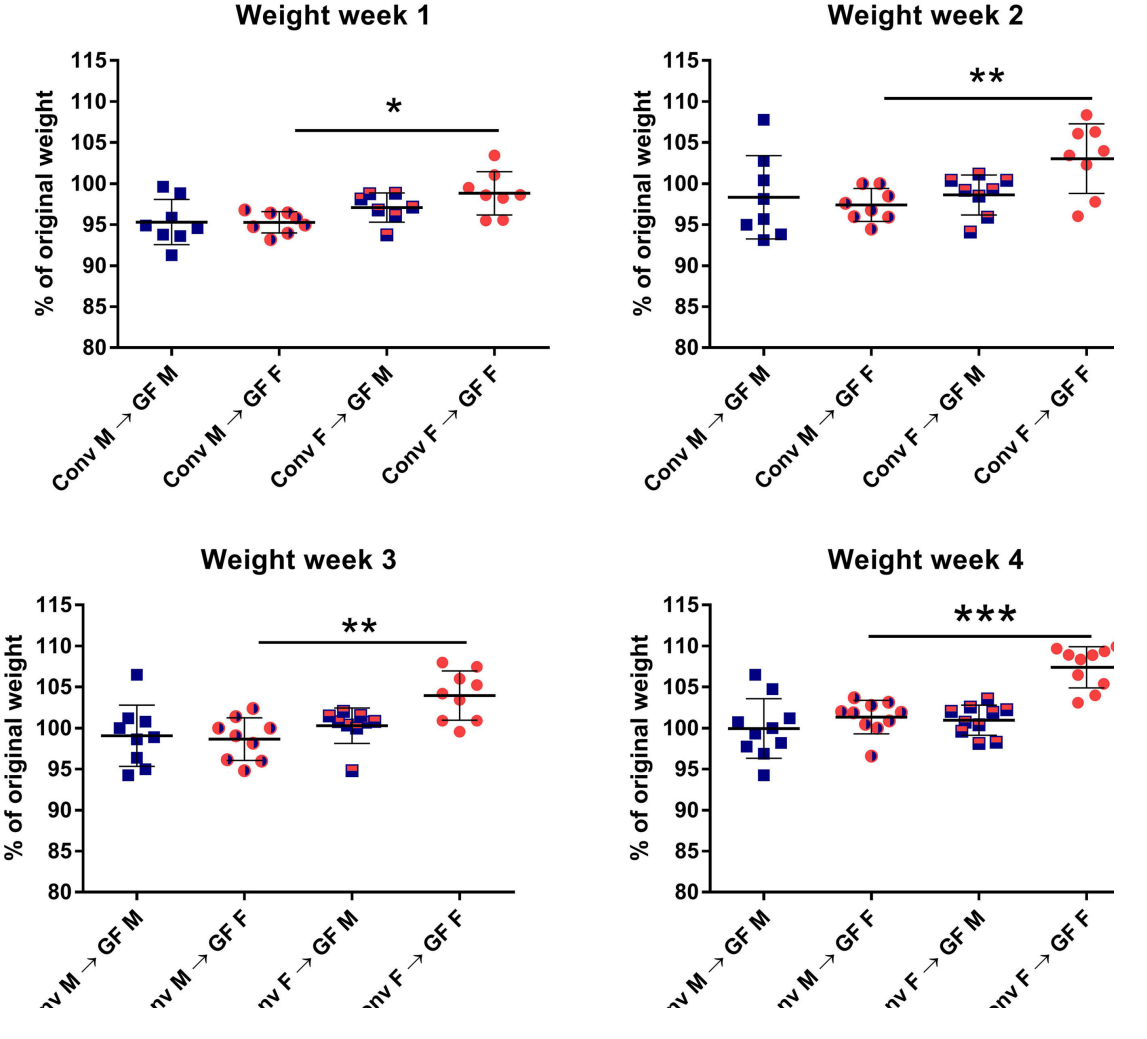
Feces of conventional (conv) males (M) or females (F) was transferred to germ-free (GF) male or female recipient mice (*n* = 10) by oral gavage. Weight was measured once a week for 4 weeks and is presented as the percentage of the original weight just before gut microbiota transfer. * indicates *p* < 0.05, ** indicates *p* < 0.01, and *** indicates *p* < 0.001.

In order to investigate how gender influences the interplay between the gut microbiota and the immune system of the host, we transferred gut microbiota from male or female conventional mice to GF mice of the same or opposing gender. After microbiota transfer, the weight of the GF recipient mice was followed for 4 weeks. All GF recipient mice lost some weight in the first week after the microbiota transfer (Figure 1). However, GF females that received male microbiota had lost significantly more weight (*p* < 0.05) compared to GF females with female microbiota. The weight of the female recipients of a male microbiota developed in a similar fashion as the male recipients of male microbiota. This weight difference remained until the end of the experiment (*p* < 0.01). A gender-dependent effect of the microbiota on weight was not observed in male GF recipients.

### Female Microbiota Enhances T Cell Precursors in Thymus but Lowers RORyt^+^ Foxp3^+^ T Cells in Male Recipients

**Figure 2:**
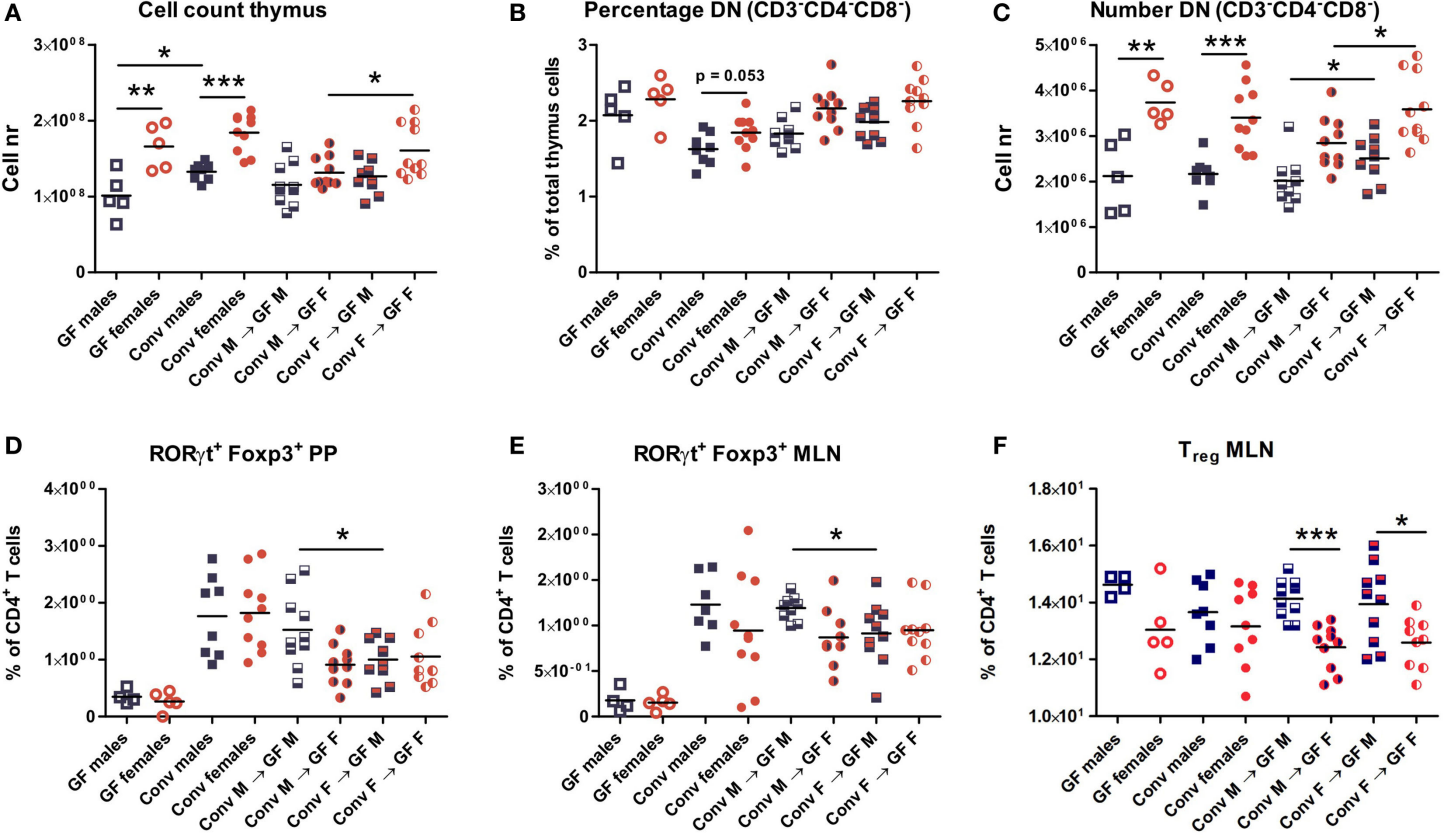
Cells from thymus, spleen, Peyer’s patches (PPs), and mesenteric lymph nodes (MLNs) were analyzed with flow cytometry. Germ-free (GF) males (M) (*n* = 5), GF females (F) (*n* = 5), conventional (conv) males (*n* = 8), and females (*n* = 10) were included as controls. Experimental groups of GF recipients of gut microbiota each contained 10 mice per group. **(A)** Total number of thymus cells. **(B)** Percentage among live thymus cells of lineage negative (CD11b, CD11c, CD19, CD45R/B220, NK1.1, and TER119) cells that were also double negative (DN) for CD4 and CD8. **(C)** Absolute numbers of thymus cells that were lineage negative (CD11b, CD11c, CD19, CD45R/B220, NK1.1, and TER119) and also DN for CD4 and CD8. **(D)** Percentage of RORyt^+^Foxp3^+^ cells among CD4^+^ T cells in PPs. **(E)** Percentage of RORyt^+^Foxp3^+^ cells among CD4^+^ T cells in MLN. **(F)** Percentage of CD25^+^Foxp3^+^ regulatory cells (Treg) among CD4^+^ T cells in MLN. * indicates *p* < 0.05, ** indicates *p* < 0.01, and *** indicates *p* < 0.001.

Four weeks after microbiota transfer the mice were sacrificed and cells were isolated from the thymus, spleen, PPs, and MLNs to study the effect of the gut microbiota transfer on T cell development and differentiation. Conventional and GF mice of both genders were included as controls. Male conventional mice had a higher number of thymic cells than germ-free males (*p* < 0.05). In addition, both conventional and GF females had a higher number of thymic cells (*p* < 0.01) compared to males (Figure 2A). Intriguingly, female recipients of female microbiota also had significantly more thymic cells (*p* < 0.05) than female recipients of male microbiota (Figure 2A). There was no gender-dependent effect of the microbiota on thymic cells in male recipients.

Early T cell precursors in the thymus are DN for CD4 and CD8, after which they become double positive for CD4 and CD8, and finally develop into cells single positive for one of these molecules (25). Frequencies of DN T cell precursors tended to be higher in females (Figure 2B). Moreover, absolute numbers of DN T cell precursors were significantly higher (*p* < 0.001) in conventional and GF females compared to males (Figure 2C). This higher number of DN T-cells was present in female microbiota recipients (both male and female) compared to recipients of male microbiota (*p* < 0.05). Taken together these results suggest that the gut microbiota influences T cell development in a gender-dependent manner.

To study the impact of the gut microbiota on T cell differentiation, the composition of the different CD4^+^ Th subsets were studied in the PPs, MLN, and the spleen. The frequency of the recently described RORγt^+^ Foxp3^+^ population (26) was much lower in the PPs (Figure 2D) and MLN (Figure 2E) of GF mice. Moreover, transfer of male microbiota led to a significantly higher percentage of RORγt^+^ Foxp3^+^ cells in PPs and MLN (*p* < 0.05) compared to male recipients of female microbiota (Figures 2D–E). Frequencies of conventional Tregs were also higher (*p* < 0.001) in male recipients compared to female recipients, but this was not dependent on the microbiota (Figure 2F). No differences between the experimental groups were observed for the Th1, Th2, or Th17 subsets in any of the organs tested (Figure S1).

### Microbiota Influences Antibody Production in a Gender-Specific Manner

**Figure 3:**
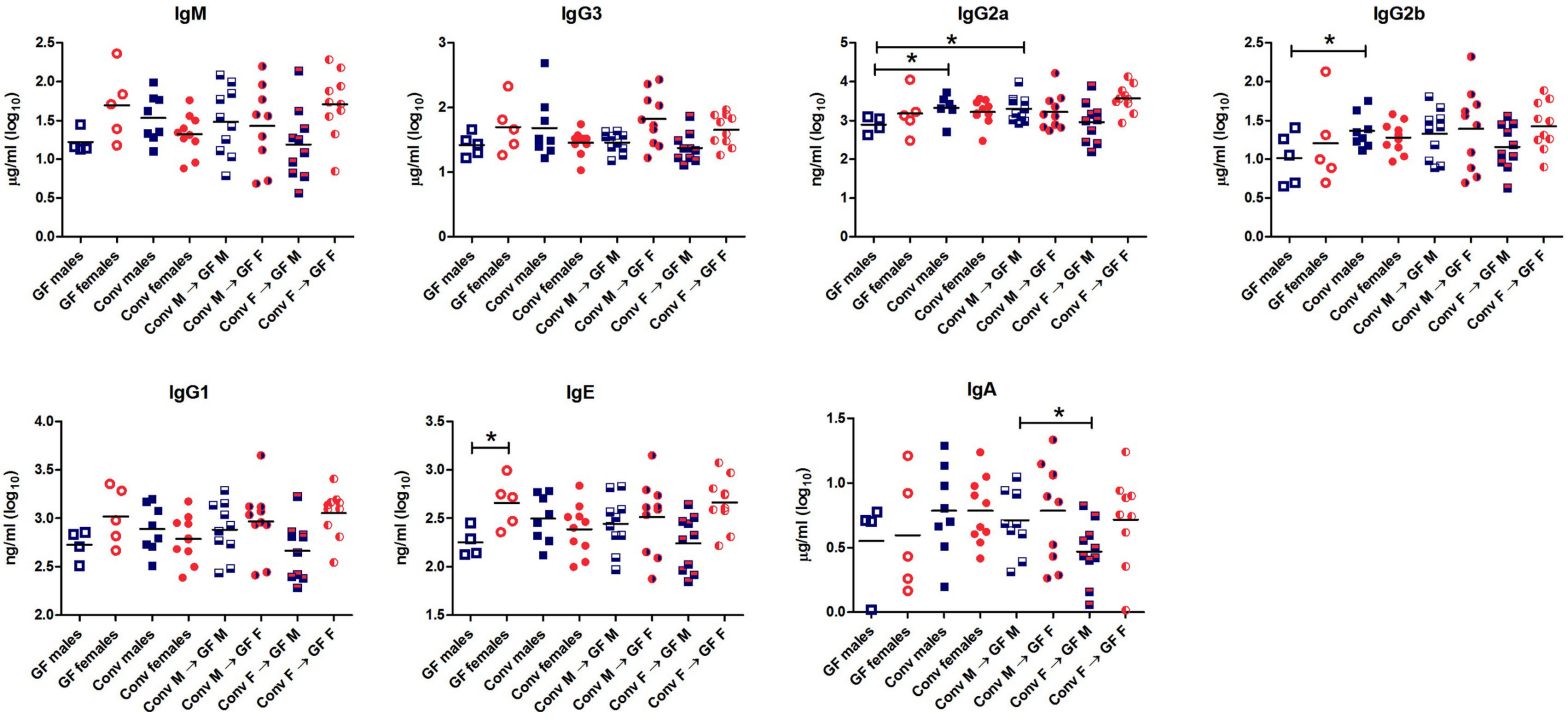
Total levels of IgM, IgG3, IgG2b, IgG2a, IgG1, IgE, and IgA were measured with luminex in serum of germ-free (GF) males (M) (*n* = 5), GF females (F) (*n* = 5), conventional (conv) males (*n* = 8), conventional females (*n* = 10), and the GF recipient male or female mice that received the gut microbiota from the same or opposite gender (*n* = 10 per group). * indicates *p* < 0.05.

To study the effect of microbial colonization on antibody production, total levels of the different antibody isotypes were measured in the serum (Figure 3). GF females tended to have higher levels of IgM (*p* < 0.1) and significantly higher levels of IgE (*p* < 0.05) compared to GF males. These data suggest that females intrinsically produce higher levels of these antibody isotypes compared to males independent of the microbiota. Moreover, conventional females did not have different levels of any antibody isotype compared to GF females, which might suggest that in females gut microbiota did not influence antibody production in the systemic compartment. On the other hand, conventional males tended to have higher levels of IgM (*p* < 0.1) and significant higher levels of IgG2a and IgG2b (*p* < 0.05) compared to GF males (Figure 3). Thus gut microbiota seemed to influence antibody production in males but not in females. Interestingly, the female microbiota significantly (*p* < 0.05) lowered IgA levels compared to male microbiota in male GF recipients.

### Microbiota-Independent and Dependent Differences in Gender-Specific Immunity

To determine microbiota-dependent and -independent differences in gender-specific immunity in the intestine, we performed genome-wide gene expression analysis of the ileum with microarray. Ileum was chosen because of its primary intestinal role in immune signaling (6). Data were analyzed with Ingenuity Pathway Analysis (IPA), only focusing on genes that were significantly differentially expressed (*p* < 0.05, fold change >1.2 or <−1.2).

**Figure 4:**
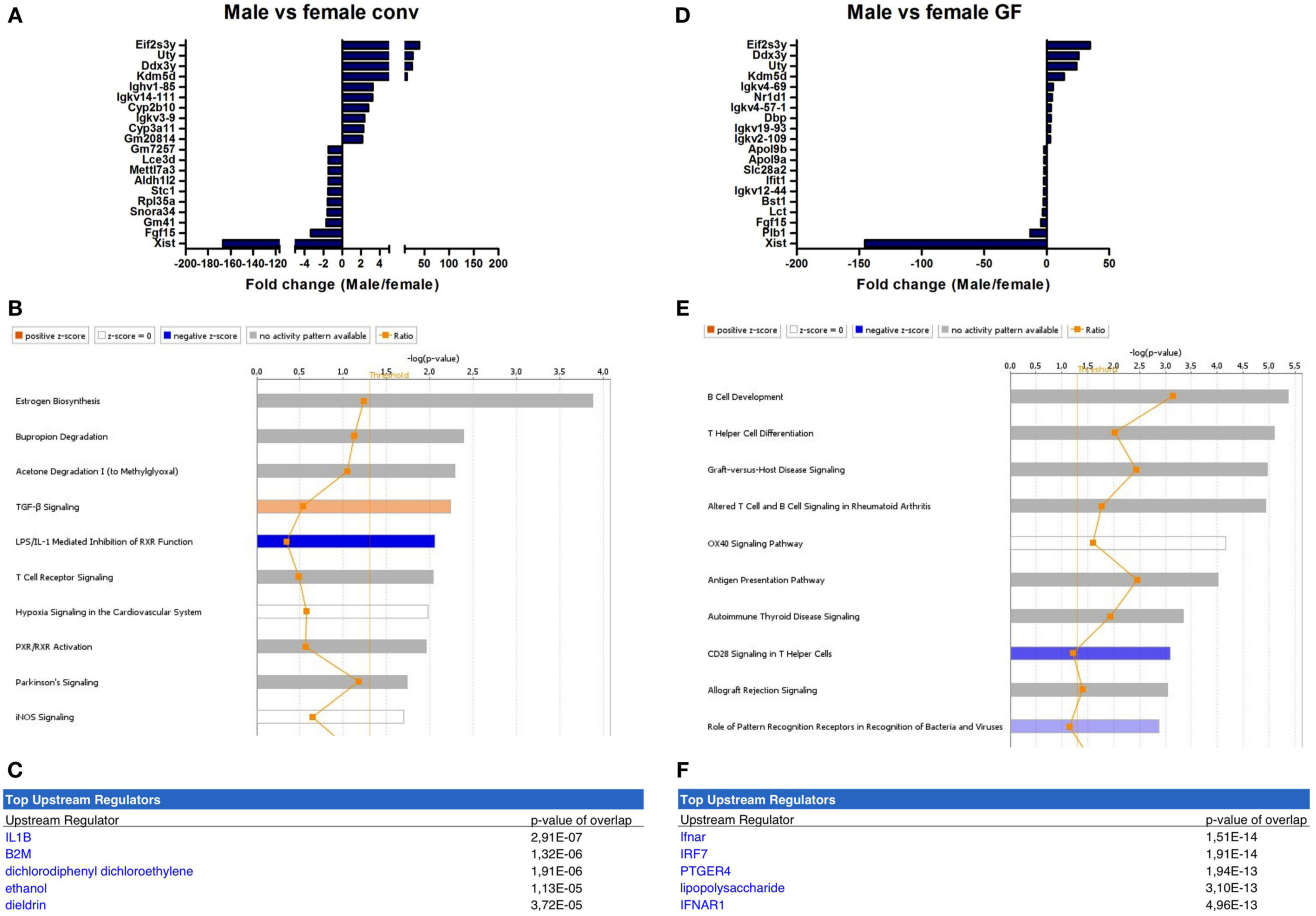
Microbiota-independent differences in immunity between males and females. Whole-genome gene expression in the ileum of male and female conventional (conv) mice (*n* = 5 per group) or male and female germ-free (GF) mice (*n* = 4) was assessed with Affymetrix GeneChip Mouse Gene 1.1 ST arrays. Genes that were significantly differentially expressed (*p* < 0.05 and fold change >1.2) between males and females, either conventional or germ free were analyzed with Ingenuity Pathway Analysis. **(A)** Top 10 of most upregulated genes and top 10 of most downregulated genes by comparing conventional males with conventional females. **(B)** Canonical pathways that were most significantly affected by gender in conventional mice. **(C)** Most significantly predicted upstream regulators of the pathways affected by gender in conventional mice. **(D)** Top 10 of most upregulated genes and top 10 of most downregulated genes by comparing GF males with GF females. **(E)** Canonical pathways that were most significantly affected by gender in GF mice. **(F)** Most significantly predicted upstream regulators of the pathways affected by gender in GF mice.

The microbiota-independent differences were studied by comparing gender-specific differences in GF animals and conventional mice. A number of genes were identified that were exclusively expressed in females (Xist) or in males (Eif2s3y, Uty, Ddx3y, Kdm5d). This difference was similar between conventional (Figure 4A) and GF mice (Figure 4D). Canonical pathways that were differentially regulated in different genders in conventional mice included “estrogen biosynthesis,” but also immune pathways such as “TGF-β signaling” and “T cell receptor signaling” (Figure 4B). The prediction by IPA of the pro-inflammatory cytokine IL-1 β as the most significant upstream regulator further confirmed the prominent differences in immunity between conventional males and females (Figure 4C).

Strikingly, the canonical pathways most significantly affected by gender in GF mice were all immune-related (Figure 4E). These pathways for example included “B cell development,” “Th cell differentiation,” and “antigen presentation pathway.” This difference between GF males and females could be due to differences in the regulation of type I interferon (IFN) production, since the receptor for type I IFN (IFNAR) and a transcription factor involved in type I IFN production (IRF7) were predicted as most significant upstream regulators by IPA (Figure 4F). In conclusion, some of the gender-dependent differences in immunity are not dependent on the gut microbiota, but are also present in GF mice.

To determine gender-specific microbiota effects, we compared ileal microarray data of (i) GF female recipient mice that received male or female microbiota and (ii) GF male recipient mice that received male or female microbiota.

**Figure 5:**
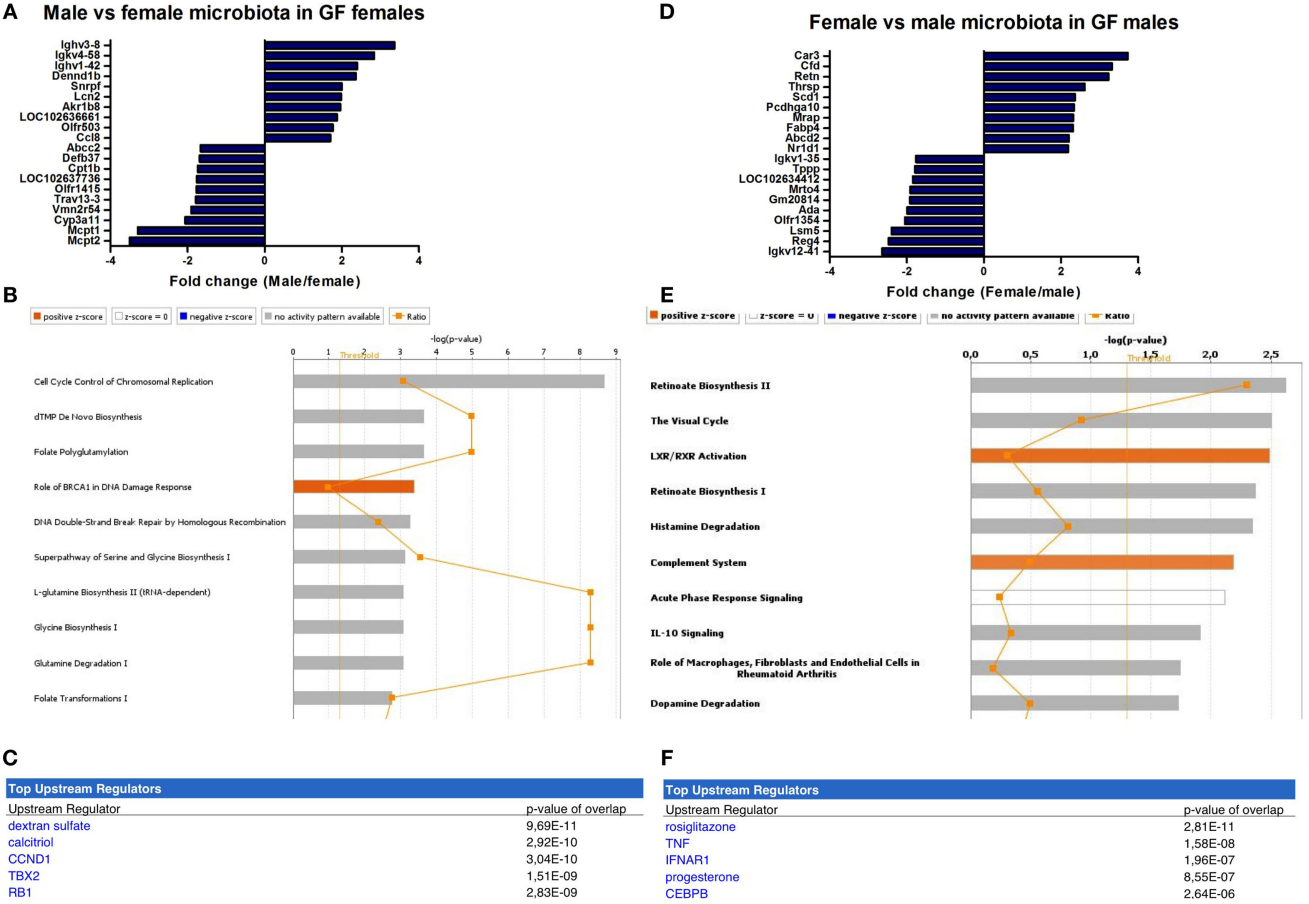
Microbiota-dependent differences in gender-specific immunity. Whole-genome gene expression in the ileum of male and female germ-free (GF) mice (*n* = 5) that received the gut microbiota from male or female conventional mice was assessed with Affymetrix GeneChip Mouse Gene 1.1 ST arrays. Genes that were significantly differentially expressed (*p* < 0.05 and fold change >1.2) between recipients of male and female microbiota were analyzed with Ingenuity Pathway Analysis. **(A)** Top 10 of most upregulated genes and top 10 of most downregulated genes by comparing male microbiota with female microbiota in GF female recipients. **(B)** Canonical pathways that were most significantly affected by gender of the microbiota in GF female recipient mice. **(C)** Most significantly predicted upstream regulators of the pathways affected by gender of the microbiota in GF female recipient mice. **(D)** Top 10 of most upregulated genes and top 10 of most downregulated genes by comparing female microbiota with male microbiota in GF male recipients. **(E)** Canonical pathways that were most significantly affected by gender of the microbiota in GF male recipient mice. **(F)** Most significantly predicted upstream regulators of the pathways affected by gender of the microbiota in GF male recipient mice.

In female recipients, the male microbiota upregulated several genes involved in the immune response, including a number of immunoglobulin variants, Dennd1b and Lcn2 (27–29) (Figure 5A). Female microbiota in GF females resulted in different effects. Here, the most highly upregulated genes were proteases Mcpt1 and Mcpt2, which are expressed by intestinal mucosal mast cells (30). Most canonical pathways that were significantly affected by the gender of the microbiota in GF recipients mice were involved in DNA repair and the cell cycle (Figure 5B). Moreover, the most significant predicted upstream regulator of these pathways was dextran sulfate (Figure 5C), which is a well-known inducer of experimental colitis (31).

In male recipients of female microbiota, the most highly upregulated genes included genes with known roles in the immune response, but also metabolism, including Cfd and Retn (32, 33) (Figure 5D). The most highly upregulated genes by the male microbiota in GF male recipients included Reg4, an antimicrobial protein recognized for its role in host–microbiota interactions (34), and ADA, which is crucial for the development of the immune system (35). The most significantly affected canonical pathways by the gender of the microbiota in GF male recipients included “complement system,” “acute phase response signaling,” and “IL-10 signaling” (Figure 5E). The most significant predicted upstream regulators were rosiglitazone, which is used for the treatment of type 2 diabetes (36) and the pro-inflammatory cytokine TNF-α (Figure 5F).

**Figure 6:**
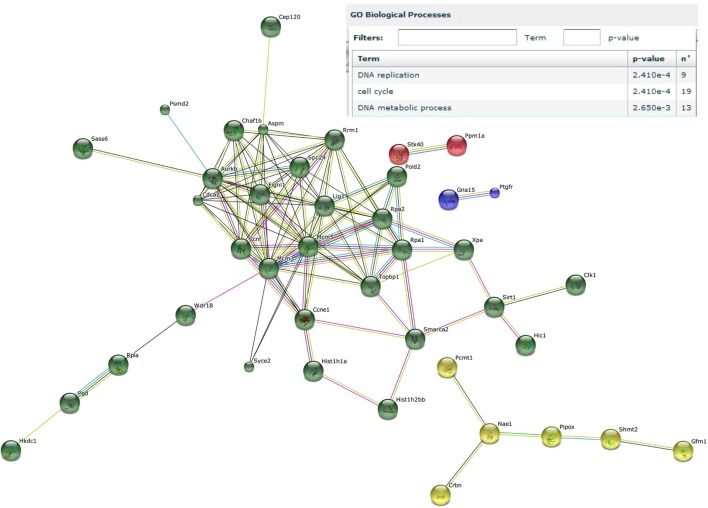
Male microbiota influences DNA replication and the cell cycle. Whole-genome gene expression in the ileum of male and female germ-free (GF) mice (*n* = 4), or male and female GF recipient mice (*n* = 5) that received the gut microbiota from male or female conventional mice was assessed with Affymetrix GeneChip Mouse Gene 1.1 ST arrays. Only genes that were significantly differentially expressed (*p* < 0.05 and fold change >1.2) were included in the analysis. Genes that were specifically affected by the male microbiota were selected as follows: affected by male microbiota in male GF recipients compared to male GF controls AND affected by male microbiota in female GF recipients compared to female GF controls, but NOT affected by female microbiota in female GF recipients compared to female GF controls, OR female microbiota in male GF recipients compared to male GF controls. The resulting list of genes was analyzed with the STRING database. Only genes with at least one interaction are shown. The following interactions are indicated: from curated databases (blue), experimentally determined (pink), textmining (yellow), co-expression (black), and protein homology (purple).

Next, genes that were induced by the male microbiota both in male and female GF recipients, but not by the female microbiota in neither male nor female GF recipients, were analyzed with the STRING database (37). We identified a number of gene clusters specifically induced by the male microbiota, which are significantly predicted to be involved in the gene ontology biological processes DNA replication and the cell cycle (Figure 6). A similar analysis of the response induced by the female microbiota did not reveal any clusters or pathways that were significantly affected (data not shown).

### Bacterial Groups Associated With Gender-Specific Microbiota Differences

**Figure 7:**
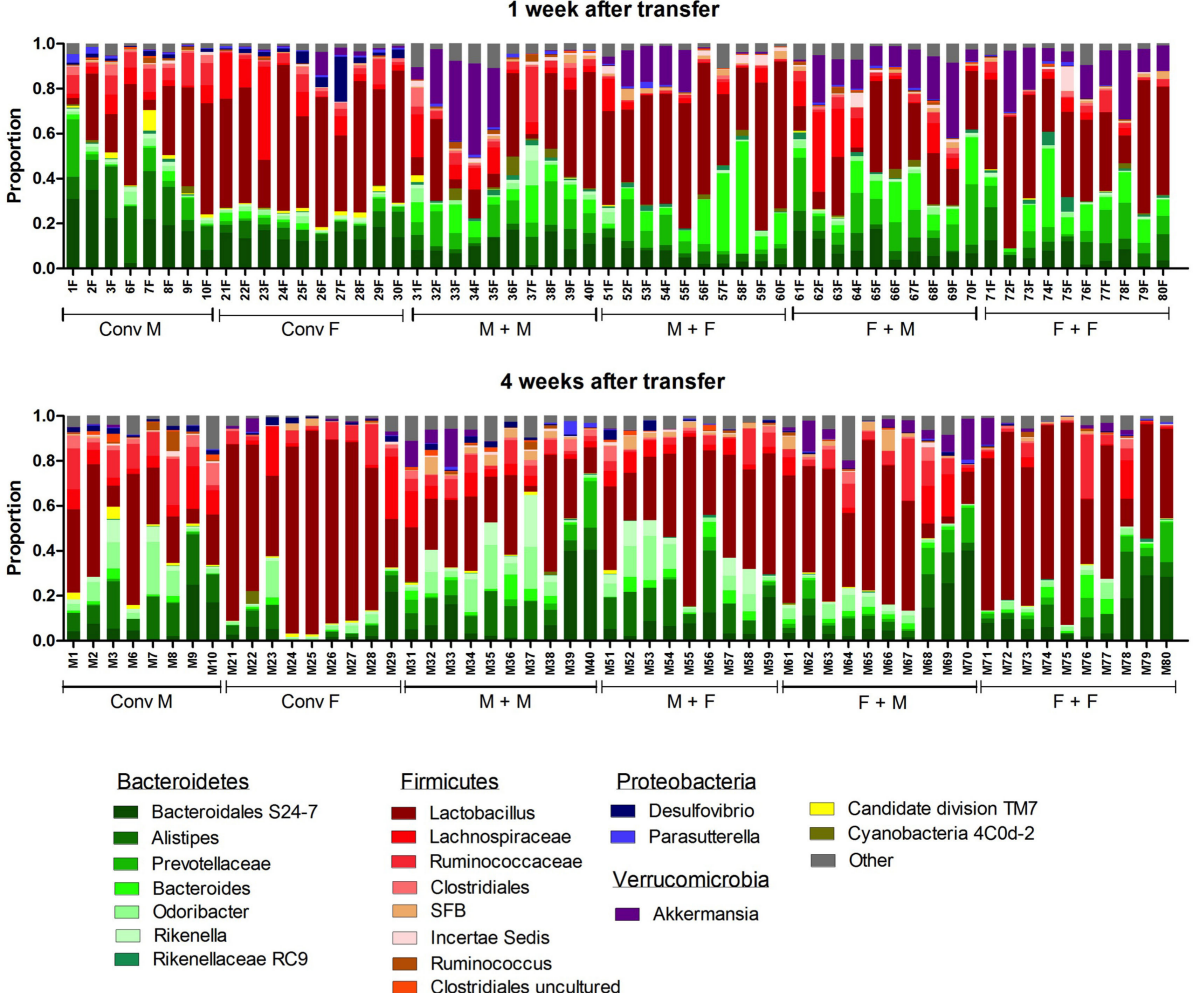
Gut microbiota composition in conventional and conventionalized mice. Fecal samples were collected from conventional (conv) males (M) (*n* = 8) and conventional females (F) (*n* = 10) at the time of transfer or 4 weeks after transfer to the germ-free (GF) recipient mice. In addition, fecal samples 1 and 4 weeks after the transfer were collected from the following groups (all *n* = 10 per group): male GF recipient mice that received male microbiota (M + M), female GF recipient mice that received male microbiota (F + M), male GF recipient mice that received female microbiota (M + F), and female GF recipient mice that received female microbiota (F + F). Gut microbiota composition was analyzed with 16S rDNA sequencing and data are presented as the relative abundance of the different bacterial groups for each individual mouse. The most highly abundant bacterial groups are indicated.

**Figure 8:**
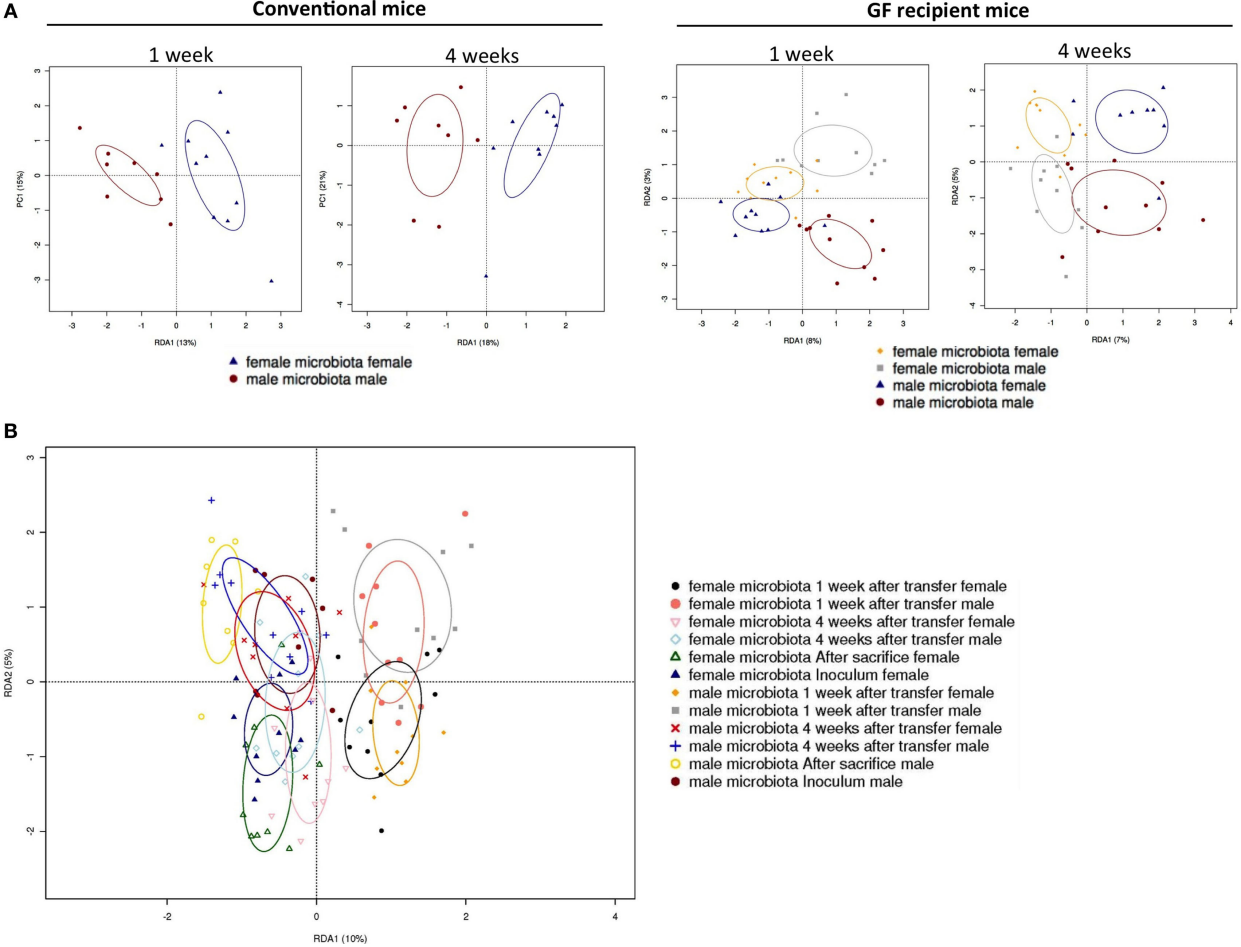
Redundancy analysis (RDA) of gut microbiota composition. Fecal samples were collected from conventional (conv) males (M) (*n* = 8) and conventional females (F) (*n* = 10) at the time of transfer or 4 weeks after transfer to the germ-free (GF) recipient mice. In addition, fecal samples 1 and 4 weeks after the transfer were collected from the following groups (all *n* = 10 per group): male GF recipient mice that received male microbiota (male microbiota male), female GF recipient mice that received male microbiota (male microbiota female), male GF recipient mice that received female microbiota (female microbiota male), and female GF recipient mice that received female microbiota (female microbiota female). Gut microbiota composition was analyzed with 16S rRNA gene sequencing. **(A)** RDA of gut microbiota composition of conventional males and females at the time of transfer (first panel), or conventional males and females 4 weeks after transfer (second panel), or GF recipient mice 1 week after transfer (third panel), or GF recipient mice 4 weeks after transfer (fourth panel). **(B)** RDA of gut microbiota composition of all groups combined.

To investigate how the gut microbiota composition changes over time in the recipient mice, composition of the gut microbiota of the different experimental groups was analyzed with 16S rRNA gene sequencing. From the male and female conventional mice, we analyzed feces at the time of transfer or 4 weeks after transfer (Figure 7). From the GF recipient mice, we analyzed feces 1 week after transfer or 4 weeks after transfer. A fundamental question to answer was whether the gut microbiota evolves into a community similar to the donor or whether it adapts to its host. Redundancy analysis (RDA) at the genus level confirmed that gut microbiota composition was different between male conventional mice and female conventional mice, since the samples separated into two distinct clusters at the time of transfer and 4 weeks after transfer (Figure 8A). One or four weeks after transfer of male or female microbiota to GF mice of both genders, four separate clusters of each experimental group could be distinguished (Figure 8A). These results imply that both the donor and host shape the gut microbiota community, leading to a unique composition in each experimental group. To further investigate how the gut microbiota communities from the different groups were related to each other, we performed another RDA analysis including all the groups and time points (Figure 8B) Surprisingly, this analysis revealed that for both genders the microbiota first adapted to the gender of the recipient 1 week after the transfer, but 4 weeks after the transfer gut microbiota composition was similar to the gender of the donor, regardless the gender of the recipient.

**Figure 9:**
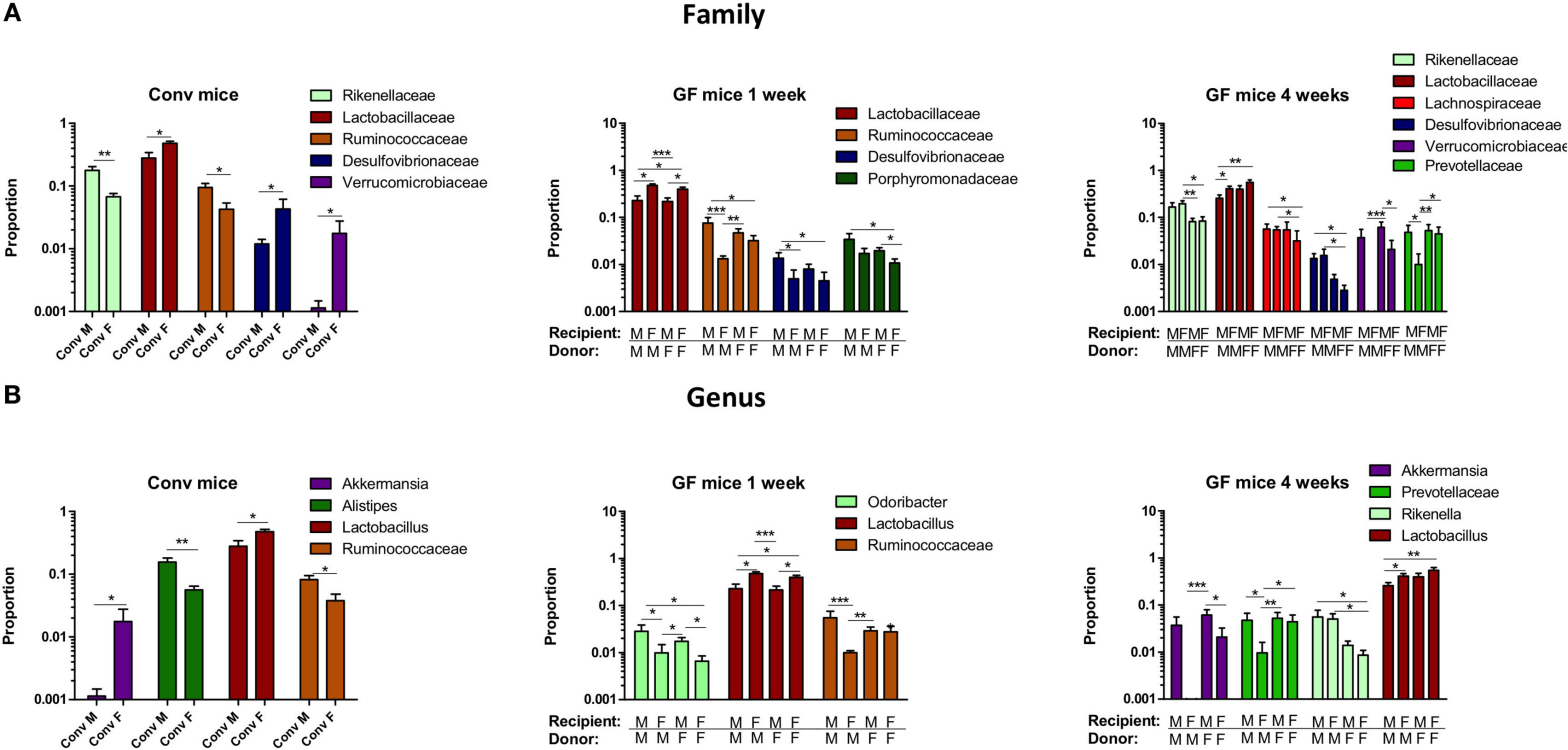
Gender-specific differences in gut microbiota composition. Fecal samples were collected from conventional (conv) males (M) (*n* = 8) and conventional females (F) (*n* = 10) at the time of transfer or 4 weeks after transfer to the germ-free (GF) recipient mice. In addition, fecal samples 1 and 4 weeks after the transfer were collected from the following groups (all *n* = 10 per group): male GF recipient mice that received male microbiota (male microbiota male), female GF recipient mice that received male microbiota (male microbiota female), male GF recipient mice that received female microbiota (female microbiota male), and female GF recipient mice that received female microbiota (female microbiota female). Gut microbiota composition was analyzed with 16S rRNA gene sequencing. **(A)** Bacterial families that were significantly different in abundance in the different experimental groups. **(B)** Bacterial genera that were significantly different in abundance in the different experimental groups.

To study more specifically the bacterial groups that were potentially responsible for the observed differences in immune responses, we investigated which bacterial groups had a significant difference in abundance at the family level (Figure 9A) or genus level (Figure 9B). Conventional females had higher abundance of Desulfovibrionaceae, Lactobacillaceae (*Lactobacillus* at the genus level), and Verrucomicrobiaceae (*Akkermansia* at the genus level), whereas conventional males had higher abundance of Ruminococcaceae and Rikenellaceae (*Alistipes* at the genus level). One week after transfer to GF recipients, the strong influence of the gender of the recipient was very clear, since all significant differences were dependent on the gender of the host. Female recipients had higher abundance of Lactobacillaceae (*Lactobacillus* at the genus level) and male recipients had higher abundance of Desulfovibrionaceae, Ruminococcaceae, and Porphyromonadaceae (*Odoribacter* at the genus level). Four weeks after transfer, most differences were still dependent on the gender of the host, but there were also some donor-dependent differences. For example, lactobacilli were still more abundant in female recipients, whereas *Akkermansia* and *Prevotellaceae* were more abundant in male recipients. However, in particular in GF female recipients, there was also a clear influence of the gender of the donor. In these mice, *Rikenella*, Lachnospiraceae, and Desulfovibrionaceae were increased after transfer of male microbiota, but Prevotellaceae was increased after transfer of the female microbiota.

## Discussion

Several studies have demonstrated that gender influences gut microbiota composition (8–11) and the immune system (1–5). However, whether the differences in gut microbiota composition between males and females are a cause or consequence of gender-specific differences in the immune system is not known. Here, we demonstrate that some characteristics of gender-specific immune differences can be induced by the gut microbiota.

Some differences in the immune system between males and females were also present in GF mice, suggesting these differences were not dependent on the gut microbiota. In particular, the type I IFN pathway was enhanced in the intestine of GF females. This can explain several gender-effects reported in literature. It has been shown that plasmacytoid DCs of females produce higher levels of IFN-α after TLR7 activation compared to males (38, 39). Furthermore, type I IFN has been shown to regulate intestinal homeostasis (40). For example, mice with conditional deletion of the type I IFN receptor (IFNAR1) in intestinal epithelial cells had an altered gut microbiota composition, which promoted epithelial hyperproliferation and experimental colitis-associated cancer (41). Therefore, it is conceivable that our observed enhanced type I IFN production in female intestines contributed to the selection of a gender-specific gut microbiota composition.

Interestingly, several bacterial groups, such as *Alistipes, Rikenella*, and Porphyromonadaceae, known to expand in the absence of innate immune defense mechanisms were overrepresented in the male microbiota in our study before or after transfer to GF mice. This is corroborated by the observation that Rikenellaceae and Porphyromonadaceae were more abundant in the gut microbiota of MyD88-deficient mice also lacking adequate innate immunity (42). Moreover, NOD2-deficiency has been shown to cause dysbiosis in mice, including a higher abundance of *Rikenella*, which induced transmissible colitis and colorectal cancer (43). Finally, in mice deficient in IL-10 and the antimicrobial peptide Lipocalin-2, *Alistipes* was shown to flourish and was sufficient to induce colitis and tumorigenesis in IL-10 deficient mice (44). Notably, Lipocalin-2 was among the genes that were most highly upregulated in female recipients of male microbiota in our study. In summary, these results may indicate that a lower innate immune response in the gut of males promoted growth of specific bacteria with the potential to promote intestinal inflammation.

Several lines of evidence suggest that in our study the male microbiota induced more gut inflammation after transfer. Female recipients lost significantly more weight after receiving male microbiota compared to female microbiota. Weight loss is a sign of discomfort and a hallmark of dextran sulfate sodium (DSS)-induced colitis (31). Interestingly, DSS was also most significantly predicted as upstream regulator when comparing gene expression in the ileum of female recipients of male or female microbiota. We also observed that DNA repair and cell cycle pathways were specifically induced by the male microbiota. This response could be due to increased inflammation induced by the male microbiota, since inflammation was previously shown to promote DNA damage and subsequent carcinogenesis in the colon (45).

The gut microbiota also influenced T cell precursors in the thymus and T cell differentiation in PPs and MLN in a gender-specific manner. We found that the thymus of females contained more cells compared to males. This corroborates the observation that thymus size is influenced by sex hormones and is larger in females (46). However, to our knowledge, we are the first to show that thymus size is also influenced by the gut microbiota in a gender-specific manner. In addition, the female microbiota induced less RORyt^+^Foxp3^+^ T cells in PPs and MLN in male recipients compared to the male microbiota. This cell population has recently been shown to be induced by the microbiota and to inhibit Th2-associated pathology (26). Thus, the female microbiota might be less efficient in preventing allergies. Indeed IgE-mediated food allergies are known to be more prevalent in adult females (47).

Together our results suggest that microbiota-independent gender immune differences contribute to the selection of a gender-specific gut microbiota composition, which in turn further drives gender immune differences. Therefore, gender should be considered in the development of strategies to target the gut microbiota in different disorders.

## Ethics Statement

This study was carried out in accordance with the recommendations of FELESA guidelines and the ethical committee for animal experiments from the University of Groningen (DEC-RUG). The protocol was approved by the ethical committee for animal experiments from the University of Groningen (DEC-RUG).

## Author Contributions

FF designed the experiments and wrote the manuscript. FF, AB, TB, and BM performed the experiments. SA, FH, and HS generated and analyzed the microbiota data. CJ and MJ provided material and resources. MB generated and analyzed microarray data. HFS, MF, and PV supervised the study.

## Conflict of Interest Statement

The authors declare that the research was conducted in the absence of any commercial or financial relationships that could be construed as a potential conflict of interest.

## Supplementary Material

Figure S1 Cells from spleen, Peyer’s patches (PPs), and mesenteric lymph nodes (MLNs) were analyzed with flow cytometry. Germ-free (GF) males (M) (*n* = 5), GF females (F) (*n* = 5), conventional (conv) males (*n* = 8), and females (*n* = 10) were included as controls. Experimental groups of GF recipients of gut microbiota each contained 10 mice per group. **(A)** Percentage of RORyt^+^Foxp3^+^ cells among CD4^+^ T cells in the spleen. **(B)** percentage of CD25^+^Foxp3^+^ regulatory cells (Treg) among CD4^+^ T cells in the spleen and PPs. **(C)** Percentage of T-bet^+^ Th1 cells among CD4^+^ T cells in the spleen, MLNs, and PPs. **(D)** Percentage of Roryt^+^ Th17 cells among CD4^+^ T cells in the spleen, MLNs, and PPs. **(E)** Percentage of Gata-3^+^ Th2 cells among CD4^+^ T cells in the spleen, MLNs, and PPs.
