# Supplementary figures and images for: The Impact of Gut Microbiota on Gender-Specific Differences in Immunity
Source: Front Immunol. 2017 Jun 30;8:754. doi: 10.3389/fimmu.2017.00754 (PMC5491612; doi:10.3389/fimmu.2017.00754)

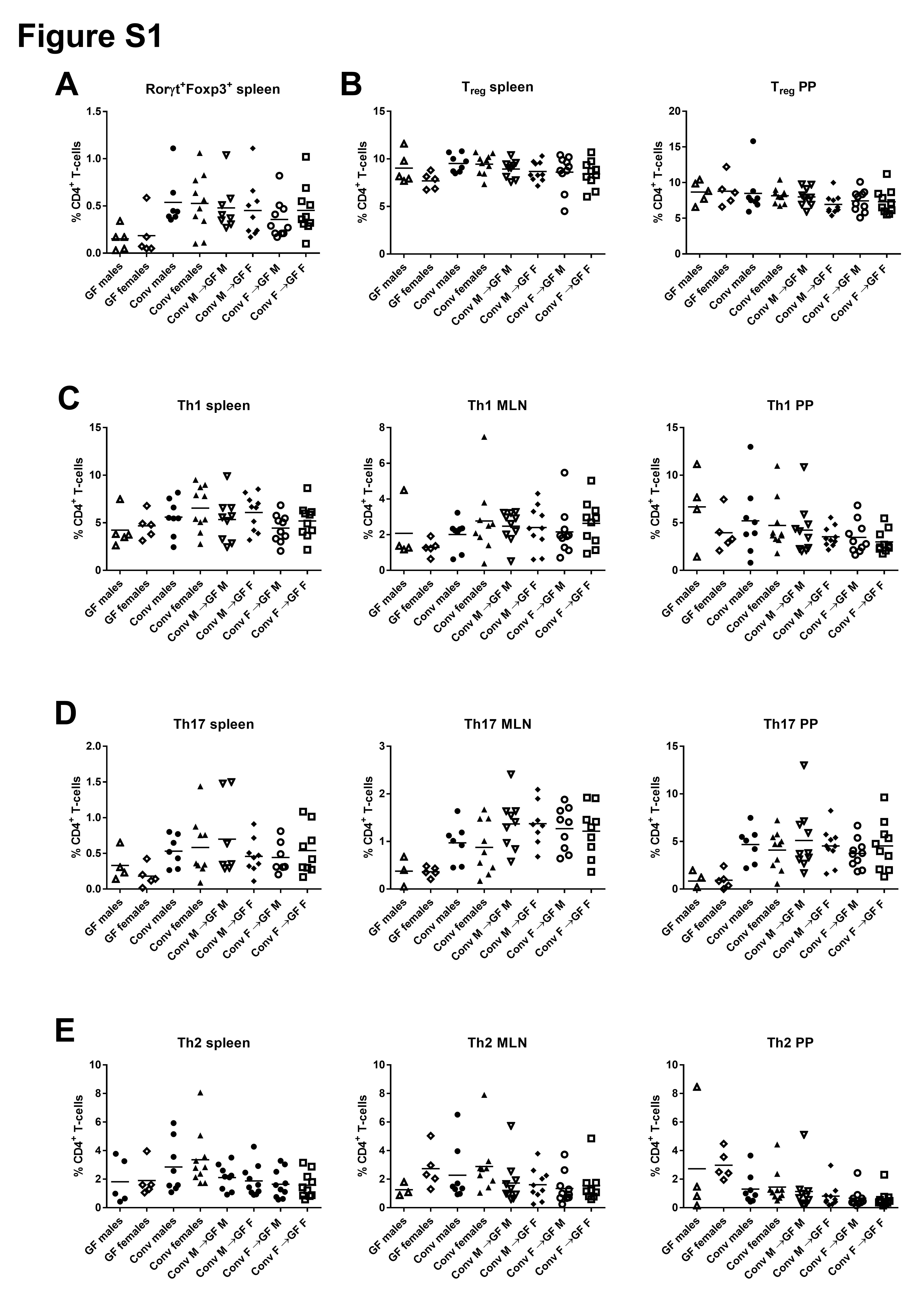

Supplement: Figure S1 — Cells from spleen, Peyer’s patches (PPs), and mesenteric lymph nodes (MLNs) were analyzed with flow cytometry. Germ-free (GF) males (M) (n = 5), GF females (F) (n = 5), conventional (conv) males (n = 8), and females (n = 10) were included as controls. Experimental groups of GF recipients of gut microbiota each contained 10 mice per group. (A) Percentage of RORyt+Foxp3+ cells among CD4+ T cells in the spleen. (B) percentage of CD25+Foxp3+ regulatory cells (Treg) among CD4+ T cells in the spleen and PPs. (C) Percentage of T-bet+ Th1 cells among CD4+ T cells in the spleen, MLNs, and PPs. (D) Percentage of Roryt+ Th17 cells among CD4+ T cells in the spleen, MLNs, and PPs. (E) Percentage of Gata-3+ Th2 cells among CD4+ T cells in the spleen, MLNs, and PPs. [file Image_1.JPEG]
